# ‘Browning’ the cardiac and peri-vascular adipose tissues to modulate cardiovascular risk

**DOI:** 10.1016/j.ijcard.2016.11.074

**Published:** 2017-02-01

**Authors:** Peter Aldiss, Graeme Davies, Rachel Woods, Helen Budge, Harold S. Sacks, Michael E. Symonds

**Affiliations:** a The Early Life Research Unit, Division of Child Health, Obstetrics and Gynaecology, School of Medicine, University Hospital, University of Nottingham, Nottingham, UK, NG7 2UH; b VA Greater Los Angeles Healthcare System, Endocrinology and Diabetes Division, and Department of Medicine David Geffen School of Medicine, Los Angeles, CA 90073, USA

**Keywords:** Epicardial adipose tissue, Perivascular adipose tissue, Brown adipose tissue, CVD

## Abstract

Excess visceral adiposity, in particular that located adjacent to the heart and coronary arteries is associated with increased cardiovascular risk. In the pathophysiological state, dysfunctional adipose tissue secretes an array of factors modulating vascular function and driving atherogenesis. Conversely, brown and beige adipose tissues utilise glucose and lipids to generate heat and are associated with improved cardiometabolic health. The cardiac and thoracic perivascular adipose tissues are now understood to be composed of brown adipose tissue in the healthy state and undergo a brown-to-white transition i.e. during obesity which may be a driving factor of cardiovascular disease. In this review we discuss the risks of excess cardiac and vascular adiposity and potential mechanisms by which restoring the brown phenotype i.e. “re-browning” could potentially be achieved in clinically relevant populations.

## Highlights

• Epicardial, paracardial and thoracic perivascular adipose tissues resemble BAT at birth.
• Despite ‘whitening’ in early life these depots remain metabolically active and potentially thermogenic into adulthood.
• Obesity induces further ‘whitening’ and inflammation in these depots likely driving the atherogenesis.
• Maintaining or inducing the brown phenotype in these depots could prevent atherosclerotic disease.

## 1 Introduction

Excess adiposity is a major independent risk factor for cardiovascular disease (CVD) [1], [2] and the associated metabolic syndrome. Pathological changes in white adipose tissue with obesity directly contribute to both metabolic abnormalities and the atherosclerotic process [3], [4]. Visceral adiposity, compared to subcutaneous fat accumulation, is recognised to have a greater impact on cardiovascular disease (CVD) which may be due in part to its close proximity to the heart. In contrast, brown adipose tissue (BAT) is a thermogenic organ that expresses the unique uncoupling protein (UCP)1 on the inner mitochondrial membrane, enabling it to circumvent ATP production and dissipate chemical energy as heat [5]. In humans reduced BAT function is closely associated with obesity, compromised metabolic health and cardiovascular risk [6], [7], [8]. The activation of existing BAT, through the recruitment of brown adipocytes or the ‘browning’ of white adipocytes to ‘beige’ cells could be a new therapeutic target for combating cardiometabolic disease.

The purpose of this review will be to a) give an overview of the health risks of excess cardiac and vascular adipose tissues b) discuss how this may be related to a transformation from brown to white adipose tissue (“whitening”) and c) highlight potential interventions to ‘brown’ these depots with the specific intent of improving cardiovascular health.

## 2 Defining the cardiac adipose tissues

**Fig. 1:**
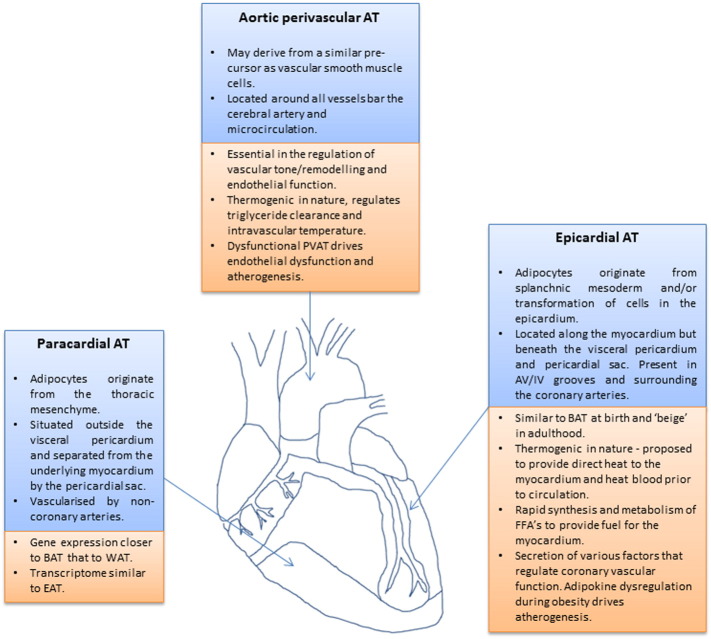
Anatomical location, physiological and pathological roles of paracardial, epicardial and perivascular adipose tissues.

Terms to describe cardiac adipose tissue vary in the literature and are used interchangeably. It is therefore important to clarify the specific anatomical location and origin of each fat depot as despite their close proximity they have distinct differences in embryological origin [9] (Fig. 1).

### 2.1 Paracardial adipose tissue

Often termed intra-thoracic [10], mediastinal [11] or pericardial, is situated on the external surface of the fibrous layer of the pericardium, vascularised by non-coronary arteries and consists of adipocytes originating from the thoracic mesenchyme [9].

### 2.2 Epicardial adipose tissue (EAT)

EAT is considered to originate from the splanchnic mesoderm, however, recently it is shown to be derived from mesenchymal transformation of cells in the epicardium [12], [13]. It is vascularised by branches of the coronary arteries. EAT is located between the myocardium and the visceral layer of the pericardium [12] accounting for ~ 20% of total heart weight [14], covering 80% of the cardiac surfaces [15] and present in the atrioventricular and interventricular grooves, within and along the myocardium and surrounding the coronary arteries [14], [16]. Importantly, there is no fibrous layer separating EAT from the underlying myocardium and coronary vessels hence the theory that EAT locally modulates CVD risk by secreting factors acting in a paracrine fashion on both cardiomyocytes and the vasculature.

### 2.3 Pericardial adipose tissue

Pericardial adipose tissue is a broad term used when referring to the total mass of both epicardial and paracardial adipose tissues.

### 2.4 Intramyocardial adipose tissue

This term is given to the adipocytes located within the myocardium itself. Classically these have been hypothesised to spill over into the myocardium from the adjacent hypertrophic EAT due to the absence of muscle fascia putatively contributing to lipotoxicity in adjacent cardiomyocytes [17]. More recently however it has been shown that intramyocardial lipid accumulation occurs when adipocytes are generated both from the developing endocardium [18] and by the differentiation of atrial cardiac mesenchymal progenitors [19].

### 2.5 Perivascular adipose tissue (PVAT)

PVAT is defined as the adipose tissue situated outside the blood vessels being structurally distinct from the adventitia and also not separated from it by a fibrous layer. Present in varying amounts around all arteries bar the cerebral artery and microcirculation [20].

## 3 Physiological roles of the cardiac and vascular adipose depots

### 3.1 Paracardial adipose tissue

Little is known about its precise role with most studies predominantly focussed on either perivascular or EAT due to their close proximity to the vasculature. Its gene expression profile is closer to that of BAT than subcutaneous adipose tissue [11] and its transcriptome is also similar to EAT [21] in men with CVD. Paracardial adipose tissue expresses a pathogenic profile characterised by increased expression of glucocorticoids and macrophage infiltration during CAD [22], [23]. Hypothetically, it may be both thermogenic and a metabolically active endocrine organ capable of contributing to systemic inflammatory processes modulating CVD progression.

### 3.2 EAT

It serves a multitude of roles essential to both survival and cardiovascular function. As the depot of fat that surrounds the coronary arteries, EAT acts in a similar fashion to PVAT providing mechanical protection during the contraction from neighbouring tissues [9], [24] such as the myocardium. Similarly, as a perivascular depot EAT plays a key role in modulating coronary vascular tone and function through the secretion of numerous vasoactive factors such as leptin [25], [26], adiponectin [27], nitric oxide [28] and angiotensin (1–7) [29] among others [20]. Metabolically, EAT has the highest rate of lipogenesis and free fatty acid (FFA) metabolism of all fat depots [30], although this was observed in adult guinea pigs and has not been replicated in other animal models or humans. EAT is hypothesised to store intravascular FFA to protect cardiomyocytes from excess exposure when raised in plasma, but also releases them to provide energy for the myocardium [30], [31]. The storage hypothesis of excess FFAs as a protective function against myocardial lipotoxicity has not been rigorously tested because this would require the coronary arteries to perfuse EAT before they penetrate the myocardium as distinct vessels which is not normally the case. Physiologically the propensity to rapidly synthesise and metabolise FFA is vital given that in humans they are the primary fuel of the myocardium [32]. EAT expresses thermogenic genes typically associated with BAT and beige adipose tissue [33], [34]. It has been proposed to provide direct heat to the myocardium conferring a survival advantage by protecting the heart during hypothermia, ischaemia or hypoxia [34]. There is no direct evidence however to suggest that these adipocytes produce heat and given their location adjacent to the contracting myocardium it is feasible they may function in non-thermogenic roles such as to alter myocardial and/or vascular redox state [33], a hypothesis supported by evidence that the ‘browning’ process modulates redox state [35] and also by the recent finding that components of the mitochondrial electron transport chain in PVAT are essential to vascular function [36]. Expression of thermogenic genes in this depot however are associated with systemic lipid homeostasis [37] and EAT may also contribute to the uptake of intravascular FFA and protect the coronary vasculature from hypertriglyceridemia associated damage. Furthermore the distribution of putatively thermogenic EAT around the coronary arteries suggest the possibility that it might be involved in maintaining myocardial temperature by heating blood in the coronaries en route to the heart [38].

### 3.3 PVAT

In healthy adults the secretory profile of PVAT is essential in the regulation and maintenance of vascular tone, remodelling and endothelial function [39]. Under pathophysiological conditions such as obesity PVAT becomes dysfunctional and compared to subcutaneous and other visceral depots expresses a higher inflammatory profile [40], releasing angiogenic factors [41] and inducing the proliferation of vascular smooth muscle cells [42] leading to endothelial dysfunction and atherosclerosis [39]. Similar to both paracardial and EAT, PVAT is phenotypically brown though their appearance depends on anatomical location such that PVAT surrounding the thoracic aorta exhibits brown characteristics and PVAT surrounding the abdominal aorta is a mixture of brown and white [43], [44], [45]. Interestingly, the ablation of PVAT in mice and the subsequent loss of its thermogenic properties impairs triglyceride clearance rendering them unable to regulate intravascular temperature [44] implicating PVAT as a key player in the maintenance of thermal homeostasis.

## 4 Excess cardiac and vascular adiposity and CVD risk

Despite Mazur et al. [46] stating in 2010 that EAT is not an independent predictor of metabolic syndrome in children and adolescents and that the prognostic value of this tissue may differ comparative to the adult population, cross-sectional epidemiological imaging data using echocardiography demonstrates a clear direct relationship between EAT and CVD risk. In obese adolescents with metabolic syndrome EAT thickness (EATT) was raised and positively correlated with fasting plasma glucose and triglycerides, HOMA-IR, carotid IMT and a range of parameters of cardiac dysfunction including left ventricular mass and myocardial performance index [47]. Similar results between lean and obese adolescents were shown by Boyraz et al. [48] who further divided the obese group into mild–moderate and severe obesity where EAT was only positively correlated with the majority of metabolic and clinical parameters in the latter group. Conversely, in both overweight and obese adolescents, EAT was significantly correlated with parameters of lipid metabolism i.e. triglycerides, HDL-C and ApoB in addition to uric acid and alanine aminotransferase indicative of a possible link between increased EAT and non-alcoholic fatty liver disease [49]. The accumulation of excess EAT has a clear association with cardiometabolic parameters in obese children and adolescents and as such makes this depot a particularly attractive target as interventions that can reduce or prevent excess cardiac adiposity in early life may be more relevant in modulating cardiovascular risk in adulthood.

The association between EAT or volume continues through to adulthood where it becomes even more pronounced and is strongly correlated with the progression and severity of CAD [50], [51], [52], [53]. The most common method to quantify EATT has traditionally been echocardiography which has some major limitations in its accuracy. For instance, typical measurements include quantifying EATT over the just one location i.e. the anterior right ventricle [50], [51], [52] or the thickness of extra-pericardial and EAT combined [53] and therefore do not constitute a true representation of the association of coronary EAT and cardiovascular risk and/or coronary atherosclerosis. Multi-detector computed tomography (MDCT) however, by way of a higher resolution and 3D views is able to accurately quantify the exact amount of EAT in various locations based on tissue density and has the ability to specify the tissue directly around the coronary arteries [10]. Similar to echocardiography studies, peri-coronary EAT (pc-EAT) is increased in CAD patients [54] and is also associated with other risk factors such as coronary artery calcium, hypertension and diabetes [55]. More detailed analysis demonstrates that vessels with coronary plaque show increased pc-EAT and that is further increased in vessels containing mixed plaques supporting the relationship of excess EAT to the atherosclerotic process. Similarly, after calculating the average thickness of pc-EAT surrounding all three coronary arteries it was shown to be thicker in those vessels with obstructive atherosclerosis [56]. These human cross-sectional studies do not prove a causal role for EAT in the pathogenesis of CAD. However, evidence for causality was generated in a pig model of coronary atherosclerosis, in which the resection of EAT from the anterior descending coronary artery ameliorated atherosclerotic plaque progression within the vessel but only at the site of adipectomy [57].

## 5 Cardiac and vascular adipose tissue dysfunction

**Fig. 2:**
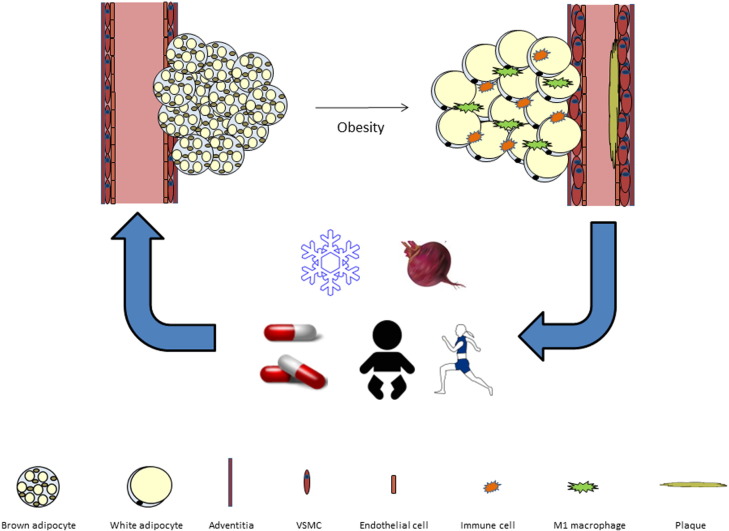
Summary figure. In the healthy state cardiac and vascular adipose tissues resemble BAT. During obesity these tissues become hypertrophic, inflammatory and dysfunctional driving endothelial dysfunction and atherogenesis. Maternal and early life (intra/extra-uterine environment), Cold exposure (SNS mediated norepinephrine release), exercise (myokine/cardiomyokine secretion), Pharmacological activation (β3 agonists and GLP1 receptor agonists) and dietary factors (nitrates/fatty acids) may modulate cardiovascular health by restoring the brown phenotype in these tissues.

It is hypothesised that pathological changes occurring in cardiac and vascular adipose tissues as they become hypertrophic from positive energy balance cause their association with CVD risk. During both ageing and chronic overnutrition, white adipose tissues expand by hypertrophy of existing adipocytes and hyperplasia of adipocyte pre-cursors [58], [59] with the concomitant recruitment of immune cells, activation of inflammatory signalling pathways, leading to adipose tissue dysfunction and a pro-inflammatory phenotype [60]. Similar to white adipocytes with the onset of obesity, multilocular lipid droplets in BAT accumulate lipid becoming hypertrophic and outstrip the vascular supply. This creates a hypoxic microenvironment leading to diminished mitochondrial function, adrenergic signalling, increased inflammation and insulin resistance [61], [62], [63]. Data from rodents [43], sheep [64], [65] and humans [11] indicate that the cardiac and vascular adipose tissues are phenotypically brown during the early stages of life and despite whitening with age retain brown characteristics in adulthood [11], [21], [33]. It could be hypothesised that further whitening of cardiac and vascular adipose tissues in obesity and the subsequent dysfunction that occurs could drive a hypoxic, inflammatory microenvironment affecting the vasculature and driving coronary atherosclerosis (Fig. 2). In support of this theory is evidence that the EAT of individuals with CAD is associated with a brown-to-white trans-differentiation characterised by significant decreases in thermogenic genes and upregulation of white adipogenesis [66]. This brown-to-white phenotype is associated with a significant increase in EAT reactive oxygen species production [66] whilst the EAT transcriptome is also characterised by markers of inflammation [67]. Furthermore, the association between EAT expression of UCP1 and circulating HDL/triglycerides suggests that functional brown adipocytes in this depot could modulate lipid metabolism in humans [37]. Given dyslipidaemia is a major contributor to atherogenesis this may be another mechanism whereby the brown-to-white switch in cardiac and vascular adipose tissues drives disease progression.

Brown and beige adipose tissues have generated significant scientific interest due to their unique ability to oxidise large amounts of glucose and lipids during UCP1 mediated thermogenesis. It is now postulated that increasing brown and/or beige adipose mass and activity is a feasible target to prevent obesity and related cardiometabolic disease [68], [69]. Adult humans retain significant amounts of metabolically active BAT which is inversely associated with BMI, age and metabolic health and importantly can be activated by either cold exposure or a B3-agonist administration [6], [70], [71], [72], [73], [74]. BAT can modulate glucose and lipid homeostasis in addition to insulin stimulated glucose disposal, insulin sensitivity and diet induced thermogenesis [75], [76], [77], [78] with substantial benefits seen in the insulin sensitivity of Type 2 diabetics [79] thus highlighting its potential clinical importance. Further evidence for a beneficial role of BAT from rodent studies demonstrates that its activation corrects hyperlipidemia [80], reduces hypercholesterolemia and protects from the development of atherosclerosis [81]. Transplantation of BAT apparently, improves not only whole body metabolism but the function of the heart and other WAT depots [82], [83]. Meanwhile beige adipocytes are functionally thermogenic and their induction is also associated with metabolic benefits [84], [85] suggestive that ‘browning’ white depots may promote similar cardiometabolic benefits.

## 6 ‘Browning’ cardiac and vascular adipose tissues to reduce cardiovascular risk

Modulation of the cardiac and vascular adipose tissue to increase the proportion of thermogenic brown or beige adipocytes could be a feasible way to improve local inflammation and reduce cardiovascular risk. However whilst there are an array of methods to ‘brown’ fat in rodents, few of these are at a stage where they could be translated to the human population, thus we will discuss only those that may have an immediate clinical application.

### 6.1 Pregnancy and early life

It is now understood that both maternal health and factors during early life have a direct influence on the phenotype of offspring adipose tissues [86], [87]. We have recently shown (in press) that EAT of the human neonate (0–29 days age) is phenotypically brown consisting of multilocular, UCP1 positive adipocytes. During the progression to infancy (1–12 months) and childhood (1–8 years) EAT undergoes a transition to primarily unilocular, UCP1 negative adipocytes with only a subset in these older age-groups having discrete islands of UCP1 positive cells. Interestingly, and similar to anorexic individuals who exhibit a reduction in the thermogenic activity of BAT [88], [89] subjects underperforming in growth scores exhibited a downregulation of thermogenic gene expression in EAT. This suggests that where nutrient availability is compromised the thermogenic machinery is reduced to maintain metabolic homeostasis. Whilst it is important to remain cautious when extrapolating results from children with various comorbidities the brown-to-white transition in early life and regulation of tissue composition during nutrient scarcity supports our previous work in sheep.

**Fig. 3:**
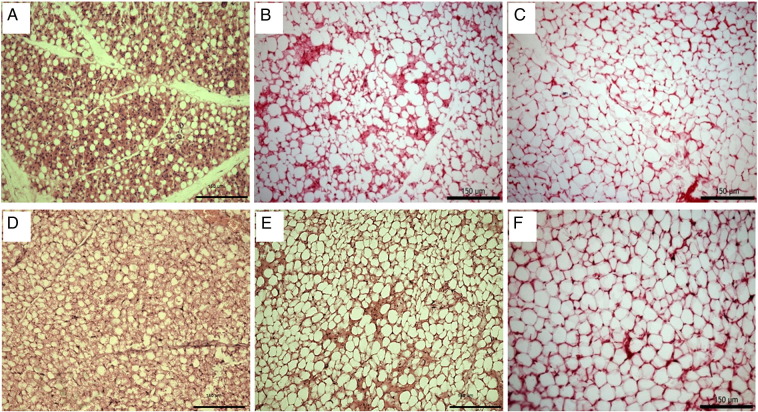
Histological brown-to-white transition of ovine paracardial adipose tissue at 1(A), 7 (B)and 28 (C) days after birth and epicardial adipose tissue at 1 (D), 7 (E) and 28 (F) days after birth. Scale bar = 150 μm

Similar to humans sheep are born with maximal, fully functional BAT to defend against hypothermia at birth making them an ideal large animal model to study the development of brown adipose tissues in early life [90]. Similar to the human neonate a clear morphological transition can be seen to occur in epicardial and paracardial AT of sheep where it resembles BAT at birth but is WAT by 28 days of age (Fig. 3). The adverse effects of the intra uterine environment during undernutrition and concomitant low birth weight have long been hypothesised to result in an increased risk of CVD [91]. Maternal nutrient restriction during late gestation in the sheep [64] down regulates the expression of thermogenic, adrenergic and mitochondrial genes in paracardial AT suggestive that reduced nutrient availability to the growing foetus compromises the thermogenic capacity of the cardiac adipose tissues. Interestingly, nutrient restriction earlier in pregnancy followed by ad-libitum feeding upregulates both UCP1 and genes involved in both white (i.e. *C/EBP*a and HoxC9) and brown (i.e. BMP7) adipogenesis [65] in pericardial AT. In rodents, the offspring of obese dams demonstrates that there is a diminished anti-contractile effect of PVAT occurring prior to both obesity and hypertension [92]. These studies highlight the importance of maternal nutrient status as it has the ability alter the thermogenic and adipogenic potential of cardiac adipose tissues whilst also programming offspring for hypertension in the absence of measureable changes in adiposity or blood pressure. Further investigations in both small and large mammals and children with CVD should be conducted to investigate the influence of maternal and early life factors on the function of these tissues. From a clinical and public health perspective it is essential to work to improve maternal and offspring health to prevent deleterious effects to the cardiac and vascular adipose tissues early in life.

### 6.2 Cold exposure

Cold exposure is the most well established activator of BAT and the browning of WAT [93], [94], which in mice also increases lipid clearance from the circulation [95] and ameliorates hyperlipidaemia [96]. Cold exposure improves the lipid profile of humans; for example, patients with hypercholesterolaemia exposed to 14 °C water over a period of 90 days had decreased LDL and total cholesterol [97]. In young healthy human volunteers undergoing controlled overnight exposure at 19 °C, improvements were seen in insulin sensitivity concomitant with an increase in BAT abundance [78].

These beneficial effects of cold exposure and BAT activation are, however, in contrast to the increased incidence of acute myocardial infarction (AMI) mortality reported in the winter months in European countries [98] and the USA [99]. Elderly individuals exposed to cold are most at risk [100] and also may lack BAT which could have a role in increased sensitivity to cold. Paradoxically increased winter mortality from AMI has also been reported in countries such as Portugal where the temperature shows relatively little seasonal variation but has higher winter AMI mortality compared to those in Northern Europe [98], indicating that factors other than temperature may also be involved. For example, it is also known that respiratory infections are increased in winter and can increase the risk of AMI [101]. Due to possible confounding factors it is difficult to elucidate the mechanism between cold exposure and possible adverse or beneficial effects on CVD risk in epidemiological studies.

Associations between cold exposure, BAT activity and atherosclerosis have been examined in controlled conditions using animal models but have reported conflicting results. A possible mechanism for cold exposure and increased AMI was proposed by Dong et al. who reported that in ApoE −/− Ldlr −/− mice exposed to 4 °C, atherosclerotic plaque growth and instability increased but was not observed with UCP1 deletion [102]. However these mice lack functional hepatic lipid clearance and cold exposure improves lipid profile in APOE*3-Leiden·CETP mice where hepatic lipid clearance is conserved [81]. A more recent study has reported however that ApoE −/− mice have increased atherosclerosis at thermoneutrality (30 °C) compared to 22 °C [103]. This raises interesting questions about the severity of cold challenge and CVD risk. It is known that mild cold exposure is sufficient to activate human BAT [78] and could therefore be activated without possible adverse effects occurring in severe cold. Therefore the beneficial adaptations to cold challenge with BAT activation still remain topics that warrant further investigation and particular caution in clinical populations with manifest CVD.

### 6.3 Pharmacological activation

Very few of the pharmacological agents used in pre-clinical research to induce browning are at a stage where they could be used in clinical studies. Fortunately, however, there exist two which are in use clinically at present and have recently been shown to induce a brown phenotype in WAT. The first of these is a new selective β3-agonist (Mirabegron) developed using cloned human β3 receptors that is currently licenced in the UK for the treatment of incontinence [104]. Previous β3-agonists mimic the effects of cold-exposure and activate beige adipocytes [93], [105] in animal models but only produce short-term improvements in heat production, insulin sensitivity and fat oxidation in humans [106], [107], [108], [109]. These discrepancies between efficacy are due to differences in receptors, pharmacokinetic properties and bioavailability between species [110] with various undesirable off-target effects on the cardiovascular system reported [111]. When given to BAT-positive healthy males Mirabegron acutely activates BAT thermogenesis and increases resting metabolic rate [112] though the dose used was four times (200 mg vs. 50 mg) that recommended for alleviating symptoms of overactive bladder and was associated with increased heart rate and both systolic and diastolic blood pressure. Efficacy of this agent at lower doses and during chronic administration still needs to be determined. Should a safe dose be established that can ‘brown’ adipose tissues it would become a good candidate to induce browning of cardiac and vascular adipose tissues by pharmacological means.

Glucagon-like peptide 1 (GLP-1) agonists Exenatide and Liraglitude are currently in clinical use for the management of hyperglycaemia in type 2 diabetes. They have been shown in both animal studies and during post-hoc analysis of phase-3 studies, as well as in the randomised double-blinded prospective Leader trial [113] to have benefits on the cardiovascular system and, in the Leader trial, major adverse cardiac events. In mice it has recently been shown that the metabolic benefits of GLP-1 agonists may occur in part through the activation of BAT and the ‘browning’ of WAT depots [114], [115], [116]. When delivered through intracerebroventricular injection, GLP-1 [114] and its analogue exendin-4 [115] increase BAT thermogenesis, mediated via an increased uptake of TG-derived FA's and plasma glucose in addition to browning WAT, effects which may occur by activation of hypothalamic AMPK [116]. Similar results have been demonstrated when GLP-1 agonists have been administered peripherally [117], [118], [119] with the browning of WAT suggested to occur via upregulation of SIRT1 [120]. Whilst these effects remain to be confirmed in humans it is feasible that GLP-1 agonists could be suitable candidates to induce browning of visceral adipose tissues.

### 6.4 Exercise

Exercise is a key modulator of cardiometabolic health [121] and elicits a number of benefits on adipose tissues including a reduction in cell number/size and inflammation [122], upregulated angiogenesis [123] and mitochondrial biogenesis [124]. In recent years it has emerged that another mechanism by which exercise improves metabolic health in rodents is by the browning of WAT whereby myokines, produced during muscular contractions, are secreted into the circulation and act in an endocrine manner on adipose tissues [125], [126]. A number of these factors are also secreted from cardiomyocytes and we speculate that these ‘cardiomyokines’ act on local cardiac and vascular adipose tissues to induce ‘browning’ and modulate cardiovascular health. Of these secreted factors, FGF21 is understood to be a potent ‘browning’ agent in rodents though its significance in humans is a topic of much debate [127]. FGF21 however is induced following exercise [127], secreted by cardiomyocytes [128] and regulates cardiac physiology [129]. It is therefore feasible that this cardiomyokine acts in a paracrine manner on EAT to induce a brown phenotype and modulate cardiovascular health. Similarly, though the subject of much debate, irisin [130] is an exercise induced PGC1-α dependent myokine that induces the browning of WAT [126], [131] whilst meteorin1, a PGC1-α4 regulated myokine induces a brown phenotype in WAT by promoting IL4/IL13 production from eosinophils and alternative M2 macrophage activation [125]. Interestingly both irisin and meteorin1 are produced by cardiac tissue and the pericardial connective tissue [132], [133]. If these are further upregulated post-exercise it is feasible that they could modulate the phenotype of the local adipose depots. Natriuretic peptides are classically secreted cardiac factors well known for their role in modulating cardiovascular homeostasis and browning adipose tissues [134] which are also upregulated post-exercise [135], [136]. The existence of a paracrine axis between beige EAT as the target and natriuretic peptides released from the atria and ventricles into the ventricular blood and then the aorta and coronary arteries seems possible but remains to be proven. Other factors that may play a role include IL-6 [137] and the metabolite lactate which is significantly increased during exercise and has recently been postulated to brown WAT to modulate tissue redox state [35]. In summary, there are an array of factors postulated to induce browning which are secreted from cardiac tissues and may act on the local adipose tissues to improve their phenotype. The effect of increasing physical activity prior to cardiac surgeries on the function of these adipose depots should be investigated in future clinical studies.

### 6.5 Nutritional intervention

Diet induced thermogenesis was initially reported by Rothwell and Stock where an upregulation of UCP1, increased BAT mass and reduced energy cost of weight gain occurred in rats fed a cafeteria diet [138]. Although diet induced thermogenesis is more controversial than cold induced thermogenesis [139] there have been several reports of nutrients and dietary compounds capable of BAT activation. Interestingly, some of these are also known to have cardio-protective effects that could be speculated to involve the browning of vascular adipose tissue depots.

Dietary nitrates, found in green leafy vegetables and beetroot have been found to have beneficial effects on lowering blood pressure and improving endothelial function in several human intervention studies [140], [141]. This is thought to be through the metabolism of nitrates to nitric oxide which is known to cause vasodilation of resistance vessels [140]. At least in some humans, dietary nitrates have also been found to increase platelet cyclic GMP [142], a signalling molecule involved in brown adipocyte thermogenesis and mitochondrial biogenesis [143], [144]. A recent study has found that feeding nitrates to rats and mice results in the upregulation of thermogenic and beta oxidation genes and UCP1 abundance in both white and brown adipose tissues through the cyclic GMP/protein kinase G pathway [145]. These browning effects were augmented in hypoxic conditions, similar to those in adipose tissue of obese individuals [145], which provides further promise for beneficial effects of dietary nitrate. This evidence provides the rationale for studies in humans to assess BAT activation with dietary nitrate which to date have not been conducted.

Conjugated Linoleic Acid (CLA) exists as a group of isomers of linoleic acid (C18:2n − 6), of which the two main biologically active isomers are the *cis*-9, *trans-*11 and the *trans*-10, *cis*-12. The *cis*-9, *trans*-11 isomer is naturally the most abundant (up to ~ 90% of total CLA [146]) and is found in ruminant dairy and meat products, where the *trans*-10, *cis*-12 isomer makes up a small percentage (~ 0.03–1.5% of total CLA [146]). These isomers are also commercially available as a supplement, where the isomers are generally mixed in varying levels.

Animal and, to a lesser extent, human studies have shown promising results for CLA supplementation in the prevention of atherosclerotic plaque development and improvements in lipid profile [147], [148]. There have also been several studies suggesting that CLA supplementation in humans can favourably alter body composition, by reducing body fat percentage [149], [150], [151], however, other studies have not observed this [152], [153]. Clear mechanisms for these cardioprotective and body compositional effects of CLA are yet to be fully identified, and there is potential that BAT could be involved in both although there are no human studies that indicate that browning can be induced by CLA. In mice the *trans*-10, *cis*-12 isomer increases energy expenditure, which correlated with increases in UCP1 mRNA [154], [155], with other studies finding that the *trans*-10, *cis*-12 isomer alone, or as a mixed isomer with *cis*-9*, trans-*11 causes browning of WAT and increased UCP1 [156], however other studies have failed to show this [157]. Work in our laboratory has shown that suckling sheep receiving milk from a mother supplemented with dietary fatty acids, which increased concentrations of total and *cis-*9, *trans*-11 CLA, exhibited an increase in UCP1 [158]. It is possible that the observed increase in UCP1 could be caused by an increase in noradrenaline, which has been reported in mice fed a mixed CLA supplement [159] and is a known activator of UCP1 [160].

There have been some negative side effects of CLA supplementation including low grade inflammation [156], however a longer term trial in humans showed no difference in adverse events between CLA supplemented and placebo groups [151]. The variable results seen between studies could be explained by the differing concentrations and doses of individual CLA isomers administered in each study. These variations make comparisons difficult and conclusions as to the role of CLA are hard to draw. More studies using a pure isomer supplementation are needed to establish causation, and whether CLA promotes adipose tissue browning in humans.

Diets rich in omega 3 polyunsaturated fatty acids, particularly long chain eicosapentaenoic acid (C20:5 n − 3, EPA) and docosahexaenoic acid (C22:6 n − 3, DHA) from marine sources or fish oil supplementation have been shown to reduce the risk of cardiovascular disease in human epidemiological studies [161] and have beneficial effects on decreasing blood pressure [162], inhibiting the progression of atherosclerosis [163], lowering plasma triglycerides and de novo lipogenesis [164]. A recent study has found that feeding mice fish oil enriched in either DHA (DHA 25%, EPA 8%) or EPA (EPA 28%, DHA 12%) induces UCP1 in both BAT and WAT through TRPV1, although browning of vascular adipose tissues in particular was not investigated. The beige marker Tbx1 and thermogenic genes such as FGF21 were also upregulated in the inguinal WAT depot [165]. An earlier report in mice however suggested that dietary supplementation with EPA/DHA to a high fat diet decreased visceral AT mass but no change in UCP1 [166]. These differing results may be, at least in part, due to the different dietary macronutrient compositions and varying amounts of EPA and DHA fed to the mice as Kim et al. used DHA (25%, EPA 8%) or EPA (EPA 28%, DHA 12%) and Janovska et al. used 46% DHA, 14% EPA. Ambient temperature also differed between the studies as Kim et al. utilised a temperature of 23 °C whereas Janovska et al. adopted thermoneutrality (30 °C) which may affect brown adipose activation. The optimum dose of EPA/DHA and conditions to promote browning in rodents is still unknown.

A recent in vitro study has shown promising results in human primary pre-adipocytes, where treatment with EPA but not DHA caused pronounced upregulation of UCP1 and mitochondrial function in pre-adipocytes and mature adipocytes [167]. Interestingly, arachidonic acid (C20:4, n − 6) treatment upregulated the white adipocyte marker TCF21. A low dietary omega 3:6 fatty acid ratio has been associated with increased CVD risk [168], it can be speculated that an increased white adipogenesis with impaired browning due to lack of omega 3 fatty acids may play a role. The effects of these fatty acids on adipose tissue browning have not yet been determined in vivo and require further investigation.

## 7 Summary

Human cardiac and perivascular adipose tissues are phenotypically brown early in life but whiten with age and obesity, becoming dysfunctional and contributing to atherogenesis in the local vasculature. Whilst active BAT may offer protection from metabolic disease, re-inducing a brown phenotype in the cardiac and vascular adipose tissues i.e. “re-browning” may be a more direct way of reducing cardiovascular risk as it likely reduces local inflammation and hypoxia adjacent to the vascular wall thus attenuating endothelial dysfunction and the atherogenic process.

This re-browning of cardiac and vascular adipose tissues may be achieved using a variety of dietary, environmental and pharmacological strategies. Future clinical trials should be considered to investigate the effects of the most appropriate interventions on the adipose tissues prior to cardiac surgeries as has been done previously when determining the effect of various treatments on vascular and myocardial tissues [169], [170], [171], [172]. If the brown phenotype can be induced in these tissues in clinical populations it will facilitate longer studies to determine if they can attenuate the atherosclerotic process. Future pre-clinical work could be directed at a) investigating the precise role each of these depots play in driving atherogenesis and other cardiovascular diseases b) determining how manipulation of the intrauterine and early life environment affects long-term function of these depots and c) develop new methods to brown these depots in adulthood.

## Conflict of interest statement

The authors report no relationships that could be construed as a conflict of interest.
